# L-3-n-butylphthalide soft capsules in the treatment of Parkinson disease dementia: A systematic review and meta-analysis of randomized controlled trials

**DOI:** 10.1097/MD.0000000000016082

**Published:** 2019-06-14

**Authors:** Rui Zhong, Qingling Chen, Xinyue Zhang, Mengmeng Li, Weihong Lin

**Affiliations:** a Department of Neurology; b Department of Hepatology, The First Hospital of Jilin University, Chang Chun, Ji Lin Province, China.

**Keywords:** L-3-n-butylphthalide soft capsules, meta-analysis, Parkinson disease dementia

## Abstract

**Background:**

In recent years, L-3-n-butylphthalide (L-NBP) has been used for Parkinson disease dementia (PDD) to attenuate cognitive impairments in China. Therefore, we selected published and qualified clinical trials to conduct a systematic review and meta-analysis with the aim of assessing the effectiveness and safety of L-NBP in the treatment of PDD.

**Objective:**

This systematic review and meta-analysis aimed to assess the effectiveness and safety of L-NBP in the treatment of PDD.

**Methods:**

We searched PubMed, EMBASE, China National Knowledge Infrastructure, Chinese Scientific Journal Database (VIP database), and Wan-Fang Database to collect eligible articles. We calculated pooled estimates of odds ratios or the standard mean deviation with 95% confidence intervals.

**Results:**

Eight randomized controlled trials were included in our meta-analysis. Our meta-analysis showed that L-NBP combined with Western medicine (WM) had a better effect on improving cognitive dysfunction, the total effective rate, symptoms of Parkinson disease (PD), and activities of daily living function than WM alone. Regarding safety, no serious adverse events were observed in the experimental group.

**Conclusion:**

We found that L-NBP as a complementary therapy may have a positive therapeutic effect for improving cognitive dysfunction, the total effective rate, symptoms of PD, quality of life, and the related serum factors in the treatment of PDD. Furthermore, L-NBP was a safe treatment for PDD. However, the findings of our meta-analysis may be influenced by the low quality of the included studies. We highlight the need to conduct trials with higher methodological quality.

## 1 Introduction

Parkinson disease (PD) is a chronic, progressive, disabling neurodegenerative disorder that is characterized by the presence of impaired motor function and nonmotor symptoms (NMS).^[1]^ Slowness of movement, muscle rigidity, and tremor are common motor symptoms, and the relevant nonmotor complaints include cognitive changes, mood disturbances, sleep disorders, and so on.^[2–4]^ These NMS of PD have detrimental effects on quality of life in these patients and cause a growing health economics.^[5]^ Parkinson disease dementia (PDD) is one common compliant among NMS in PD patients.^[6]^ A study by Cummings^[7]^ included and analyzed 27 studies (with 4336 patients enrolled), and their findings indicated that the mean prevalence of PDD reached 40% among PD patients. The prevalence of dementia in advanced PD reached 80%, which greatly affects quality of life in patients and increases healthcare and institutionalization economic costs.^[8]^ Treatment options for PDD are limited. Cholinesterase inhibitors and memantine are 2 main current treatments for PDD to improve cognitive function. Rivastigmine is the only licensed medication for mild to moderate PDD that is currently approved by the Food and Drug Administration.^[9]^ Research on Western medicine (WM) for PDD has achieved some success; however, there is still a long way to go.

L-3-n-butylphthalide (L-NBP), a small molecule compound, was isolated from Chinese celery and cress seeds in southern China. NBP has been approved for the treatment of ischemic stroke by the Chinese State Food and Drug Administration in 2002 owing to its neuroprotective effects. The potential mechanisms of NBP regarding its neuroprotective effects include anti-oxidant and anti-inflammatory effects and the stimulation of the proliferation, migration, and differentiation of hippocampal neural stem cells. L-NBP can effectively improve cognitive function and has been regarded as a potential drug for the treatment of Alzheimer disease (AD).^[10]^

In recent years, L-NBP has been used to attenuate cognitive impairments associated with PDD in China.

Several randomized controlled trials (RCTs) were carried out to test the effect of L-NBP plus WM compared with WM alone for the treatment of PDD. Therefore, we selected published and qualified clinical trials to conduct a systematic review and meta-analysis with the aim of assessing the effectiveness and safety of L-NBP in the treatment of PDD.

## 2 Methods and analysis

All analyses in the present study were based on previous published studies; thus, no ethical approval or patient consent was required.

### 2.1 Study strategy

Two reviewers independently searched 3 major Chinese databases, which included China National Knowledge Infrastructure (CNKI), Chinese Scientific Journal Database (VIP database), and Wan-Fang Database. Then, PubMed and EMBASE were also systematically searched by two reviewers. The key terms “Ding ben tai,” “Pa jin sen chi dai,” and “Pa jin sen bing” were used in the Chinese databases. In the English databases, the key words included “L-3-n-Butylphthalide,” “L-NBP,” “Parkinson's disease dementia,” “PDD,” “Parkinson's disease,” and “PD.” We restricted the language to Chinese and English. In addition, we also reviewed the reference lists of the included studies to identify eligible articles.

### 2.2 Eligibility criteria

Inclusion criteria were as follows: type of study: RCTs exploring the efficacy and safety of L-NBP for PDD were included; restriction on language: only Chinese and English studies were included; type of participants: patients were diagnosed with PDD according to standard diagnostic criteria; intervention: RCTs examining L-NBP combined with WM versus WM alone were included; outcomes: primary outcomes included assessment of cognitive function by the Montreal Cognitive Assessment (MoCA) and Mini-Mental State Examination (MMSE). Secondary outcomes included the total effective rate, Unified Parkinson Disease Rating Scale (UPDRS), activities of daily living, and levels of C-reactive protein (CRP), recombinant human PD protein 7 (PARK7) and neurotrophic factor-3 (NT-3). The exclusion criteria were as follows: the study was a duplicate; the number of individuals in each group was not reported, and the data were insufficient; and the study type was a case report, letter, review, or conference abstract. Only RCTs providing sufficient data were included in the present meta-analysis.

The search strategy in PubMed was as follows:

(1) Parkinson's disease dementia[MeSH Terms]
(2) Parkinson's disease[MeSH Terms]
(3) (PD[Title/Abstract]) OR PDD[Title/Abstract]
(4) (1) OR (2) OR (3)
(5) (Butylphthalide[Title/Abstract]) OR ((L-3-n-Butylphthalide[Title/Abstract]) OR L-NBP[Title/Abstract])
(6) (4) AND (5)

### 2.3 Study selection

Duplicates were first excluded. Then, we screened titles and abstracts and excluded studies that obviously did not fulfill the inclusion criteria. Studies that provisionally met eligibility criteria were assessed for eligibility by examining the full text. Two reviewers (Zhong and Chen) independently checked the articles and resolved disagreements by discussion.

### 2.4 Data extraction

We collected the following data from the included studies: first author name, publication year, ages, sample size, intervention details, outcomes, and treatment period. Two investigators (Zhong and Chen) independently extracted data from eligible articles, and any discrepant judgments were resolved by joint discussion.

### 2.5 Quality assessment

The quality of the included RCTs was subjectively graded using the modified Jadad Score. An RCT was defined as high quality when the total score was at least 4 (score of 4–7). Two reviewers independently evaluated the quality of the RCTs and resolved disagreements by discussion.

### 2.6 Statistical analysis

Heterogeneity was assessed by the *P* value of the *χ*^2^ and *I*^2^ statistics, and heterogeneity was significant if the *I*^2^ statistic was >50% or the *P* value was <0.05. A meta-analysis was conducted if the outcome was reported in at least 2 studies. The detailed information as reported in the studies was used. Odds ratios (ORs) or standard mean deviations (SMDs) with 95% confidence intervals (CIs) were used to pool data, and the results were presented as forest plots, which included the contribution of each study (weight) to the overall effect. We used random-effects models to pool the results when there was significant heterogeneity in our meta-analysis; otherwise, a fixed-effects model was applied. A qualitative analysis would be carried out if a quantitative analysis could not be performed on the data. In addition, a sensitivity analysis, by removing one study at a time, was conducted to evaluate the stability of the results. A publication bias was assessed if the number of included RCTs was >10 in one meta-analysis. State 12.0 was used for this meta-analysis.

## 3 Results

### 3.1 Study search results

**Figure 1:**
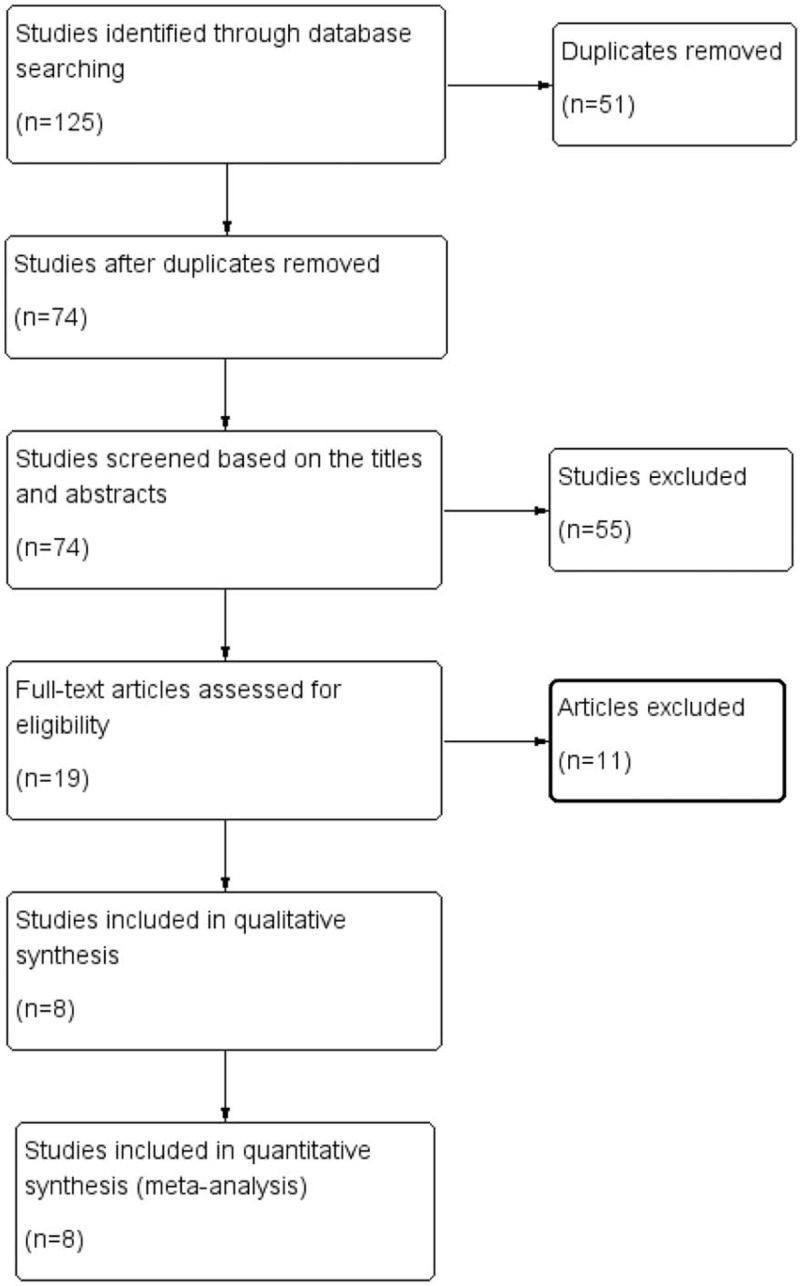
The process of identification of studies.

The search initially identified 125 articles. Seventy-four articles remained for titles and abstract screening after 51 duplicate articles were removed. Then, we excluded 55 articles that were obviously irrelevant based on the titles and abstracts. The full text of 19 studies was assessed in detail. Finally, 8 trials^[11–18]^ were included in our meta-analysis. A flowchart of the process used for selection of the studies is presented in Figure 1.

### 3.2 Characteristics of eligible RCTs

Eight RCTs, with a total of 638 participants enrolled, were eventually included in our meta-analysis. The sample size ranged from 61 to 92, and there was a median of 80 PDD patients per study. Patients in 4 RCTs received L-NBP twice per day in the EG, but the other 4 RCTS adopted a regimen of 3 times per day. The dose of L-NBP each time was 0.2 g. The treatment course ranged from 3 to 6 months. All 8 trials compared L-NBP plus WM with WM alone, and WM included donepezil hydrochloride, l-dopa, or benzhexol hydrochloride tablets. The detailed characteristics of the included RCTs are summarized in Table 1.

**Table 1:**
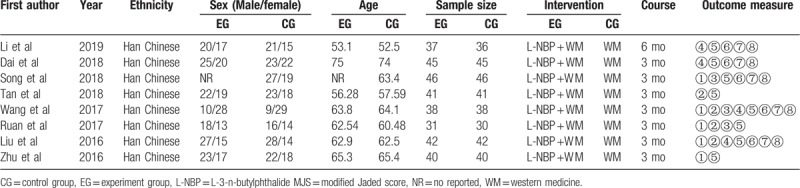
The characteristics of these included studies.

### 3.3 Quality of the RCTs

The modified Jadad Score was used to assess the quality of the eligible trials. Seven RCTs reported that they randomly generated the allocation sequence, in which 5 trials adopted the random figure table and the other 2 studies did not provide detailed information. No trials reported the blinding method. All RCTs did not provide information about the drop-out rate. The overall quality of the RCTs was considered not high, as described in Table 1.

### 3.4 Outcomes

#### 3.4.1 Primary outcomes 3.4.2 Cognitive function assessment—MoCA scores

**Figure 2:**
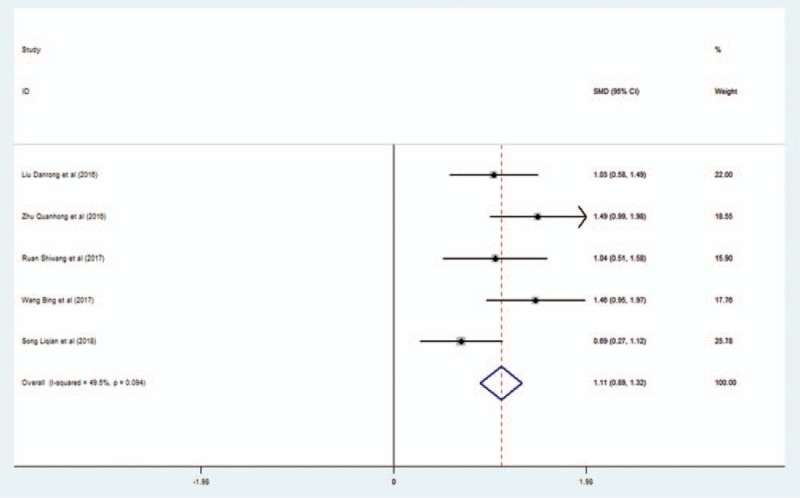
Forest plot of the effect of L-NBP plus WM versus WM on MoCA scores using the fixed effect model. L-NBP = L-3-n-butylphthalide, MoCA = Montreal Cognitive Assessment, WM = western medicine.

Five studies reported a comparison of MoCA scores between the L-NBP + WM group and the WM group. No significant heterogeneity was observed in these studies (*I*^2^ = 49.5%). Therefore, we adopted the fixed effect model. Pooled results indicated a significant difference in MoCA scores between the L-NBP plus WM group and the control group (CG) (SMD = 1.107; 95% CI, 0.893–1.321, P < 0.001) (Fig. 2).

#### 3.4.3 Cognitive function assessment—MMSE scores

**Figure 3:**
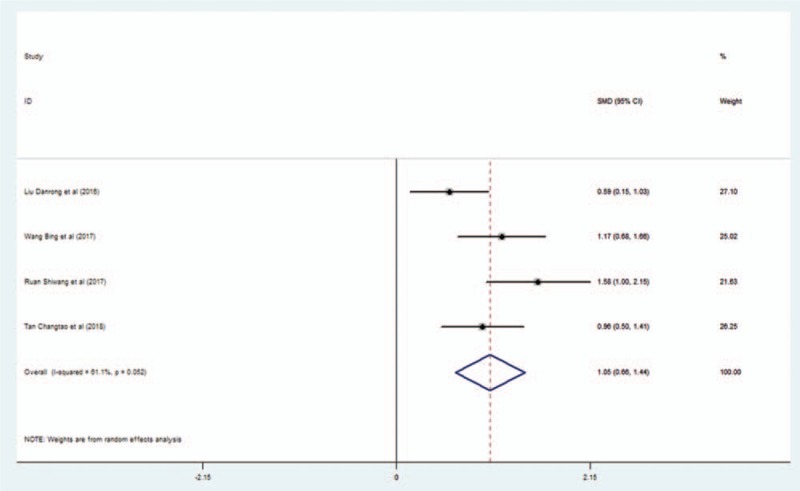
Forest plot of the effect of L-NBP plus WM versus WM on MMSE scores using the random effect model. L-NBP = L-3-n-butylphthalide, MMSE = Mini-Mental State Examination, WM = western medicine.

Four studies mentioned the effect of L-NBP plus WM on MMSE scores compared with WM alone. The random effects model was used to pool the results due to significant heterogeneity. Meta-analysis showed a better effect of L-NBP plus WM for increasing MMSE scores compared with WM alone (SMD = 1.046; 95% CI, 0.656–1.436; I^2^ = 61.1%; P < 0.001) (Fig. 3).

#### 3.4.4 Secondary outcomes

##### 3.4.4.1 The total effective rate

**Figure 4:**
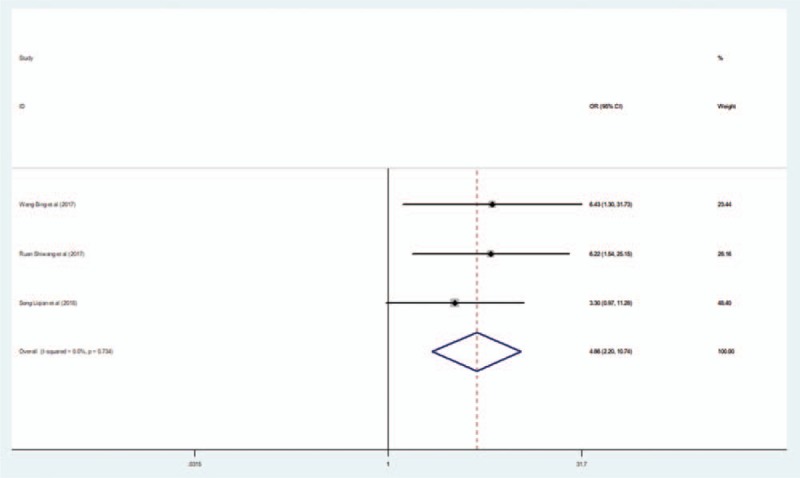
Forest plot of the effect of L-NBP plus WM versus WM on the total effective rate using the fixed effect model. L-NBP = L-3-n-butylphthalide, WM = western medicine.

Three studies reported the total effective rate. Meta-analysis indicated a significant increase for this outcome in the experimental group (EG) compared with the CG (OR = 4.856; 95% CI, 2.195–10.743; *I*^2^ = 0; *P* < .001) (Fig. 4).

##### 3.4.4.2 UPDRS

**Figure 5:**
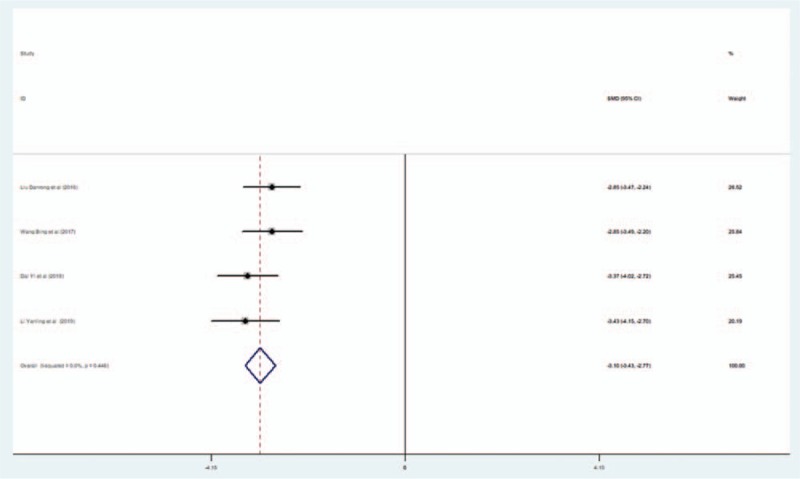
Forest plot of the effect of L-NBP plus WM versus WM on the UPDRS score using the fixed effects model. L-NBP = L-3-n-butylphthalide, WM = western medicine.

Four studies reported results from the UPDRS. The fixed-effects model was adopted to pool data towing to no significant heterogeneity (*I*^2^ = 0). The pooled results indicated that the UPDRS score was significantly decreased in the EG compared with the CG (SMD = −3.099; 95% CI, −3.426 to 2.773; *P* < .001) (Fig. 5).

##### 3.4.4.3 Activities of daily living

**Figure 6:**
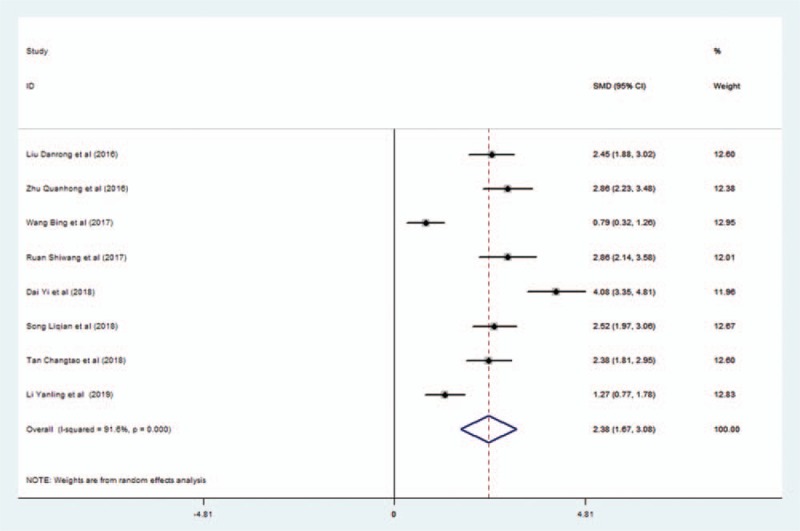
Forest plot of the effect of L-NBP plus WM versus WM on the activity of daily living using the random effects model. L-NBP = L-3-n-butylphthalide, WM = western medicine.

Eight studies reported the findings regarding activities of daily living. The random-effects model was adopted to pool data owing to significant heterogeneity (*I*^2^ = 91.6%). Pooled data showed that activities of daily living were significantly better in the EG than in the CG (SMD = 2.378; 95% CI, 1.674–3.082; *P* < .001) (Fig. 6).

##### 3.4.4.4 Serum factors

Five studies reported related serum factors, including CRP, PARK7, and NT-3. Previous studies showed that these factors were significantly associated with PD.^[19,20]^

##### 3.4.4.5 CRP

**Figure 7:**
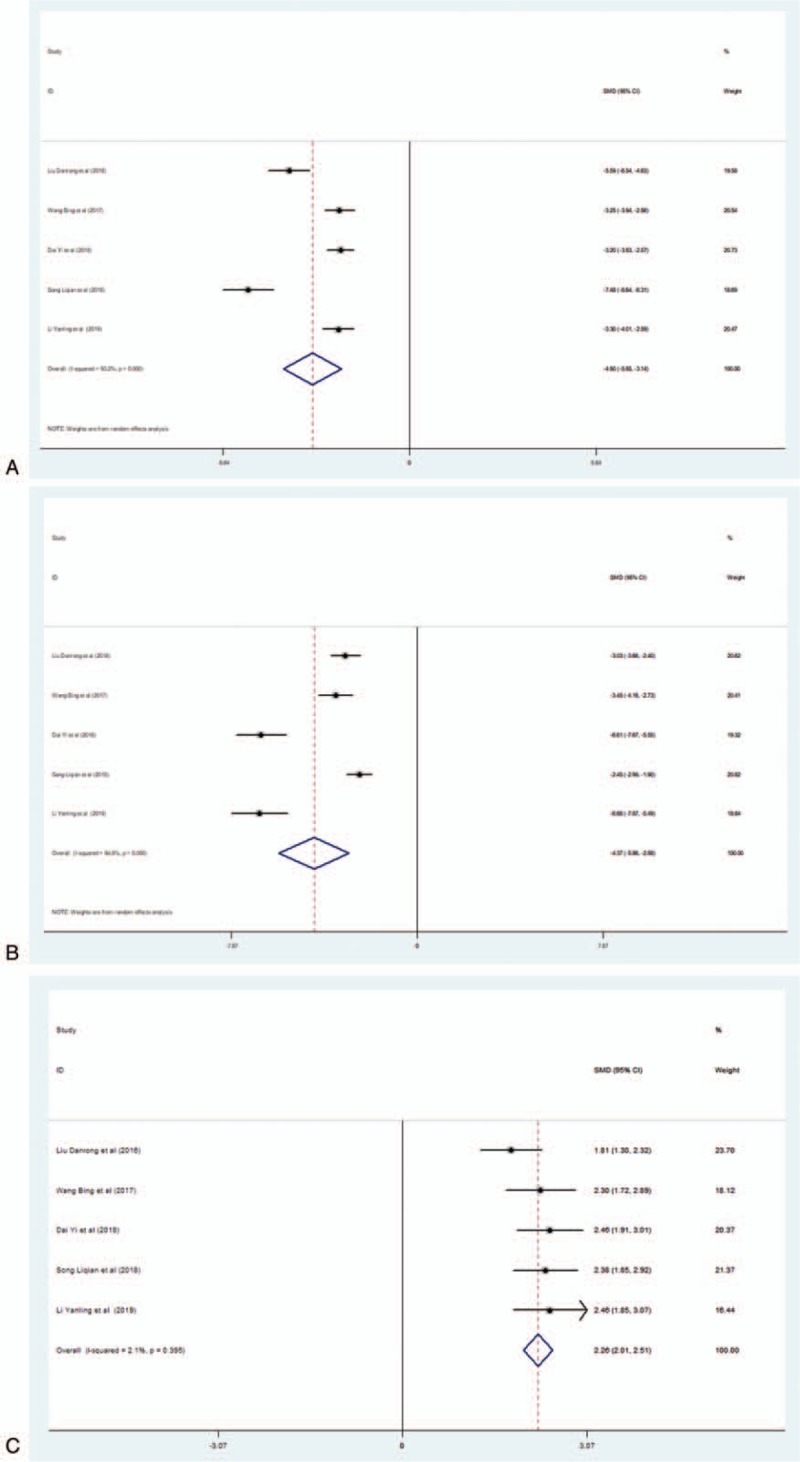
(A) Forest plot of the effect of L-NBP plus WM versus WM on CRP levels using the random effects model. (B) Forest plot of the effect of L-NBP plus WM versus WM on PARK7 levels using the random effects model. (C) Forest plot of the effect of L-NBP plus WM versus WM on NT-3 levels using the fixed-effects model. CRP = C-reactive protein, L-NBP = L-3-n-butylphthalide, WM = western medicine

There were 5 studies that measured CRP. The random-effects model was adopted to pool data due to significant heterogeneity (*I*^2^ = 93.2%). Meta-analysis indicated that there was a significant difference in the level of CRP between the EG and the CG (SMD = −4.497; 95% CI, −5.853 to 3.142; *P* < .001) (Fig. 7A).

##### 3.4.4.6 PARK7

There were 5 studies that reported on PARK7. The random-effects model was adopted to pool data because of significant heterogeneity (*I*^2^ = 94.8%). Meta-analysis indicated that there was a significant difference in PARK7 levels between the EG and the CG (SMD = −4.371; 95% CI, −5.862 to 2.881; *P* < .001) (Fig. 7B).

##### 3.4.4.7 NT-3

There were 5 studies that reported the levels of NT-3. The fixed-effects model was adopted to pool data, as there was no significant heterogeneity (*I*^2^ = 2.1%). Meta-analysis indicated that there was a significant difference in the levels of NT-3 between the EG and the CG (SMD = 2.261; 95% CI, 2.013–2.509; *P* < .001) (Fig. 7C).

##### 3.4.4.8 Safety

Only 1 study reported adverse events in the EG and CG. In this trial, gastrointestinal reactions, dizziness, nausea, or insomnia were the most common adverse events in the EG; 5 patients experienced these events including 1 had gastrointestinal reactions, 1 had dizziness, 1 had nausea, and 2 patients experienced insomnia as a problem. However, there was no significant difference in adverse events between the EG and CG. No serious adverse events were observed in the EG. The L-NBP combined with WM treatment was found to be well tolerated for PDD patients, which indicates the safety of L-NBP combined with WM. However, the other trials did not report any adverse events.

### 3.5 Sensitivity analysis

**Figure 8:**
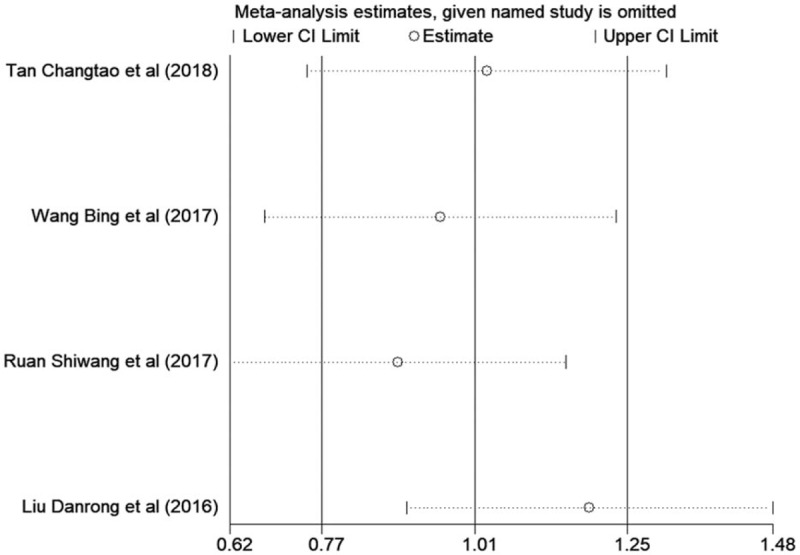
Sensitivity analysis with the MMSE. MMSE = Mini-Mental State Examination.

We carried out sensitivity analysis to assess the stability of the results. Statistically robust results were proven through sensitivity analysis (Fig. 8).

## 4 Discussion

Cognitive impairment often occurs early in PD and has been considered a risk factor for PDD. The treatment and management of cognitive function in PDD is particularly important to improve the quality of life for patients. Several previous studies based on animal models have been carried out to prove the beneficial effects of L-NBP in brain disorders, such as ischemic stroke,^[21–23]^ AD,^[24,25]^ vascular dementia,^[26]^ epilepsy,^[27]^ and PD.^[28,29]^ In recent years, L-NBP has been widely used in patients with cognitive impairment in China, including use in patients with vascular dementia, AD, and PDD. Several RCTs with the aim of assessing the efficacy and safety of L-NBP for PDD have been conducted. However, no meta-analysis on this topic has been carried out. Therefore, we selected published and qualified clinical trials to conduct a systematic review and meta-analysis to assess the effectiveness and safety of L-NBP in the treatment of PDD.

This meta-analysis explored the efficacy and safety of L-NBP in the treatment of PDD. Eight studies with a total of 638 PDD patients were included in our meta-analysis. Our meta-analysis showed that L-NBP combined with WM had a better therapeutic effect for PDD than WM alone. First, the combination use of L-NBP and WM has a notable effect on improving cognitive dysfunction in PDD patients, and the scores of the MoCA and MMSE were significantly increased in the EG compared to the CG. Second, L-NBP added to WM can increase the total effective rate, improve symptoms of PD and improve activities of daily living function more than WM alone. In addition, L-NBP combined with WM can improve the related serum factors associated with PDD, which include CRP, PARK7, and NT-3. Regarding safety, no serious adverse events were observed in the EG. In summary, the findings of our meta-analysis proved the efficacy and safety of the combination of L-NBP and WM in the treatment of PDD. To the best of our knowledge, our meta-analysis is the first to pool the results of RCTs assessing the efficacy and safety of L-NBP combined with WM in patients with PDD. Our work provided further evidence for the efficacy of L-NBP on improving cognitive function, which may be an attractive choice for PDD patients. Our meta-analysis should be helpful for clinicians who are treating PD patients with severe cognitive dysfunction.

The effect of L-NBP on cognitive impairment has been widely explored based on animal models.^[24,30–32]^ Xv et al found that L-NBP can alleviate the deficits in learning and memory in mice by activating Akt/mTOR signaling and suppressing apoptosis and autophagy. Evidence from a recent study indicated that L-NBP may have a beneficial effect on memory and learning functions by reducing oxidative stress, suppressing neuronal apoptosis and inhibiting the nuclear factor-?B signaling pathway in db/db diabetic mice.^[33]^ In addition, recent progress has indicated a neuroprotective effect of L-NBP through the rescue of dopaminergic neurons by reducing oxidative stress, which may improve the symptoms of PD.^[29]^ These findings from previous studies may help explain the results of the present meta-analysis.

Several limitations of the meta-analysis should be acknowledged. First, a meta-analysis may be biased when the literature search fails to identify all relevant studies. However, access to unpublished articles remains difficult, which might be a potential limitation of our study. Only articles written in Chinese and English were included in the present meta-analysis, which may also lead to bias. In addition, we did not perform a publication bias analysis because of the limited number of included studies. Second, the number of included studies is limited, and the sample size is small. We highlight the need to conduct more trials to test the findings of the meta-analysis. Third, the quality of the included RCTs was generally not high. Most studies had selection, performance, detection, or other types of bias. The drop-out rate was not mentioned in any included study, which is a limitation. Because tolerability evaluation as one part of the safety of LNBP is necessary to assess. Our conclusions may be influenced by these high risks of bias. In addition, it remains unclear whether there is sex difference in response to L-NBP. Subgroup analysis based on sex was not conducted because of a lack of detailed information. More RCTs with detailed information were needed to answer the question. Finally, significant heterogeneity existed among studies for some outcomes. However, we did not find the source of heterogeneity through a sensitivity analysis. Different basic characteristics of PDD patients in studies may have led to heterogeneity. The high heterogeneity in the results may also be explained by regional differences, including diet and lifestyle.

In our meta-analysis, we found that L-NBP as a complementary therapy may have a positive therapeutic effect for improving cognitive dysfunction, the total effective rate, symptoms of PD, quality of life, and the related serum factors in the treatment of PDD. Furthermore, L-NBP was a safe treatment for PDD. However, the findings of our meta-analysis may have been influenced by the low quality of the included studies. We highlight the need to conduct trials with higher methodological quality.

## Author contributions

**Conceptualization:** Rui Zhong.

**Data curation:** Rui Zhong, Qingling Chen, Xinyue Zhang, Weihong Lin.

**Formal analysis:** Rui Zhong.

**Funding acquisition:** Rui Zhong, Weihong Lin.

**Investigation:** Rui Zhong, Qingling Chen, Mengmeng Li, Weihong Lin.

**Methodology:** Rui Zhong, Xinyue Zhang, Mengmeng Li.

**Project administration:** Rui Zhong.

**Resources:** Rui Zhong, Qingling Chen.

**Software:** Rui Zhong, Qingling Chen, Xinyue Zhang, Weihong Lin.

**Supervision:** Rui Zhong, Qingling Chen, Mengmeng Li, Weihong Lin.

**Validation:** Rui Zhong.

**Visualization:** Rui Zhong, Xinyue Zhang.

**Writing – original draft:** Rui Zhong, Qingling Chen, Mengmeng Li, Weihong Lin.

**Writing – review & editing:** Rui Zhong, Weihong Lin.
